# Production-Type-Specific Spatial and Space–Time Clustering of High-Pathogenicity Avian Influenza Outbreaks in Layer and Duck Farms in the Republic of Korea, 2020–2026

**DOI:** 10.3390/ani16162543

**Published:** 2026-08-14

**Authors:** Dae Sung Yoo, Yeonsu Oh

**Affiliations:** 1 Department of Veterinary Epidemiology, College of Veterinary Medicine and Animal Medical Institute, Chonnam National University, Gwangju 61186, Republic of Korea; shanuar@chonnam.ac.kr; 2 Department of Veterinary Pathology, College of Veterinary Medicine and Institute of Veterinary Science, Kangwon National University, Chuncheon 24341, Republic of Korea

**Keywords:** high-pathogenicity avian influenza, HPAI, spatial epidemiology, space–time clustering, Cuzick–Edwards test, layer farms, duck farms, poultry farm density, Republic of Korea

## Abstract

High-pathogenicity avian influenza (HPAI) remains a recurrent threat to poultry production in the Republic of Korea, but production-type-specific clustering has rarely been assessed against the underlying farm population. This retrospective unmatched case–control point-pattern study examined spatial and space–time clustering across six epidemic seasons from 2020 to 2026. We analyzed 486 reported poultry-farm outbreak records together with a national registry of 66,499 poultry farms. The principal analyses compared 204 layer and 204 duck outbreak events with farms of the same production type using the Cuzick–Edwards k-nearest-neighbor statistic, an observed-to-expected clustering-intensity ratio, kernel relative-risk mapping, and spatial and space–time scan statistics. At k = 5, the observed-to-expected intensity ratio was 2.51 for layer events and 1.63 for duck events; this between-type contrast was descriptive because no direct inferential test was performed. Layer-event clustering remained elevated across all seasons, although the extreme 2023–2024 estimate was based on only 15 events. Duck-event clustering was more intermittent and increased in 2025–2026. Premises-level scan statistics identified a broad layer cluster with a relative risk of 3.12 and a smaller duck cluster with a higher local relative risk but far fewer farms. A layer space–time cluster occurred in 2024–2025. These findings support production-type-specific, density-aware surveillance. They do not establish farm-to-farm transmission or implicate egg-handling infrastructure, and they should be interpreted in light of the static registry, repeated-event design, and unmeasured flock size, movements, biosecurity, environmental exposure, and viral lineage.

## Simple Summary

High-pathogenicity avian influenza repeatedly affects Korean poultry farms, but outbreak maps can be misleading because poultry farms themselves are geographically concentrated. We compared outbreak events in layer and duck production with the corresponding national farm populations using complementary spatial methods. Layer-farm outbreak events had higher observed-to-expected local clustering ratios across several neighborhood sizes, whereas duck-farm clustering was more variable among epidemic seasons. The analyses also identified broad and localized high-risk areas in west-central poultry-production regions. These results describe where outbreak events were unusually concentrated relative to farms at risk; they do not identify a specific transmission route or imply greater biological transmissibility in chickens. Because the study used a single 2020 farm registry and retained repeated outbreak events at recurrent premises in event-level analyses, the magnitude of clustering should be interpreted cautiously. The findings support production-type-specific surveillance and closer investigation of farm density, flock size, service movements, biosecurity, wild-bird exposure, and viral lineage in future studies.

## 1. Introduction

High-pathogenicity avian influenza (HPAI) is caused by influenza A viruses belonging to the species *Alphainfluenzavirus influenzae* and remains one of the most consequential transboundary diseases of poultry, with effects on animal health, food security, trade, and wildlife conservation [1,2]. The scale of individual epidemics can be substantial: during the 2014–2015 epidemic in the United States, more than 50 million birds died or were depopulated, and the total economic impact was estimated at approximately USD 3.3 billion [3]. The current global H5N1 clade 2.3.4.4b panzootic has further expanded the geographic range, host range, and persistence of infection in wild birds, poultry, and mammals [4,5,6]. These developments increase the need for surveillance and control that can prioritize locations where poultry outbreaks are more likely to aggregate.

In the Republic of Korea (ROK), HPAI has caused recurrent winter epidemics since the mid-2000s. Viral incursions have repeatedly been associated with migratory waterfowl, followed in some seasons by local spread within densely populated poultry regions [7,8,9,10]. Recent genomic investigations have also documented continued introduction and reassortment of H5 clade 2.3.4.4b viruses during the 2023–2024 and 2024–2025 seasons [11]. Once a virus is introduced, the resulting farm-level pattern can reflect poultry density, production type, farm connectivity, environmental exposure, surveillance intensity, and biosecurity rather than any single pathway [7,8,12,13,14].

Local transmission has been reported in several poultry systems. In the ROK, between-farm infection risk declined steeply with distance and was associated with farm-to-farm connections and vehicle movements [7,8]. Studies in France, the Netherlands, the United States, and Canada have likewise shown that farm density, proximity, shared service networks, and contact histories can shape the spatial concentration of outbreaks [12,15,16,17,18]. These findings support surveillance that focuses on high-risk production areas, while also showing that spatial proximity alone cannot distinguish direct lateral spread from common exposure or repeated viral introductions.

Production type may modify these processes, but the contrast should not be interpreted as a simple difference in host transmissibility. Commercial duck production in Korea includes enclosed as well as more environmentally exposed systems, so contact with wild-bird-associated environments depends on housing and management. Experimental work with a representative clade 2.3.4.4b H5N1 virus demonstrated efficient infection, shedding, environmental contamination, and duck-to-duck transmission, but limited transmission among chickens under the tested conditions [19,20,21]. Consequently, stronger spatial clustering in layer production would more plausibly reflect production structure, flock size, farm density, service movements, or surveillance than greater intrinsic transmission efficiency in laying hens.

Layer production has several operational characteristics that may contribute to local aggregation. Layer farms commonly maintain large flocks over long production cycles and receive repeated visits for feed delivery, egg collection, manure removal, veterinary services, and other activities. A 2025 Japanese case–control study identified a flock size of at least 100,000 laying hens as a potential risk factor for H5N1 introduction [22]. Egg grading-and-packing operations represent one possible component of these service networks, and avian influenza virus can contaminate eggs and environmental surfaces in layer facilities [23,24]. Korean regulations therefore include separation requirements for egg grading-and-packing businesses [25]. However, the present study did not include facility coordinates, collection routes, or vehicle-sharing data and was not designed to estimate risk attributable to such facilities.

Many spatial studies map outbreak locations or model environmental risk factors, but case locations alone cannot distinguish excess outbreak clustering from the underlying aggregation of poultry farms. A case–control point-pattern approach addresses this problem by comparing outbreak events with the spatial distribution of farms of the same production type [26,27]. It therefore evaluates whether cases have more neighboring cases than expected among farms at risk, rather than treating the landscape as spatially uniform.

Few national studies have compared recent layer- and duck-farm outbreak patterns using production-type-specific background populations. Important uncertainties also remain regarding seasonal variability, the influence of recurrent events at the same premises, and whether broad regional clusters and highly localized clusters convey different epidemiological information. These questions are relevant to risk-based surveillance and to the design of studies that subsequently integrate flock size, movements, biosecurity, environmental variables, wild-bird surveillance, and viral sequences.

This study examined the spatial and space–time clustering of HPAI outbreaks in layer and duck farms in the ROK from 2020 to 2026. We used an unmatched case–control point-pattern framework to quantify event clustering relative to production-type-specific farm populations, evaluated seasonal variation and neighborhood-scale consistency, and localized premises-level high-risk foci using relative-risk mapping and scan statistics. We hypothesized that layer and duck production would exhibit distinct clustering patterns. Because the two production types were analyzed against different background populations, the observed-to-expected ratios were compared descriptively rather than through a direct between-type significance test.

## 2. Materials and Methods

### 2.1. Study Design and Overview

This retrospective unmatched case–control point-pattern study compared HPAI outbreak-event locations with the underlying distribution of poultry farms at risk in the ROK. The analysis comprised data compilation and production-type classification, outbreak-to-registry linkage, construction of production-type-specific case–control point patterns, event-level Cuzick–Edwards analyses, exploratory kernel relative-risk mapping, premises-level Bernoulli spatial scans, season-stratified analyses, and a layer-farm space–time permutation scan.

### 2.2. Data Sources

#### 2.2.1. HPAI Outbreak Data

We compiled 486 HPAI outbreak records reported in poultry premises in the ROK between November 2020 and April 2026, spanning six epidemic seasons from 2020–2021 to 2025–2026. Data were obtained from the Korea Animal Health Integrated System (KAHIS). Each record included the report date, poultry species or production type, H5 subtype, and point coordinates for the affected premises. An outbreak record was defined as an officially reported HPAI-positive poultry premises-event in the national animal disease surveillance and response system. Multiple records could therefore occur at the same registered premises in different epidemic seasons.

Outbreak records were grouped into layer (*n* = 204), duck (*n* = 204), and other poultry (*n* = 78) categories. Layer farms comprised commercial egg-producing chicken farms. The registry did not provide a consistently harmonized subdivision of meat, breeder, and layer duck operations across the study period; these were therefore combined as duck farms. Other poultry included broiler, breeder, native chicken, and minor poultry types. Main analyses focused on layer and duck production, and the heterogeneity within the duck and other-poultry categories is acknowledged as a limitation.

Each record was assigned to an epidemic season from November of the first calendar year through April of the following year, consistent with the principal seasonal pattern of poultry outbreaks in the ROK. The six periods were 2020–2021, 2021–2022, 2022–2023, 2023–2024, 2024–2025, and 2025–2026.

#### 2.2.2. National Poultry-Farm Registry

The background population at risk was derived from a national poultry-farm registry dated 1 October 2020. The registry included 66,499 premises and contained farm identifiers, production type or species, flock size at the registry date, and point coordinates. Of these premises, 1159 were classified as layer farms and 769 as duck farms. Flock size was not modeled because only the single baseline value was available, whereas flock inventories may change materially within and among epidemic seasons; using the 2020 value as a six-year time-varying exposure could therefore create additional misclassification. A harmonized breed variable was not available for adjustment.

The October 2020 registry was the most complete national farm-location dataset available for defining the population at risk and preceded the first included epidemic season. It was used as a fixed spatial background for all seasons. Farm opening, closure, relocation, and changes in production type or flock size could not be updated. Such temporal misclassification may differ between layer and duck production if their turnover patterns differed; this possibility was considered when interpreting seasonal and production-type contrasts. The data sources, study periods, dataset sizes, and roles in the analysis are summarized in Table 1.

### 2.3. Data Preprocessing and Coordinate Projection

Outbreak and registry records were screened for production type, report date, and coordinate validity. Each premises was represented as a point at its registered coordinate; polygon centroids or administrative-area centroids were not used. Coordinates were projected from WGS84 longitude-latitude to the Korea 2000 Unified Coordinate System (EPSG:5179), and all distance-based analyses used projected metric coordinates.

Because outbreak and registry records originated from separate administrative datasets, the distance from each outbreak location to the nearest registered farm of the same production type was calculated. A 1 km tolerance was used to reconcile administrative coordinate discrepancies; it was a data-linkage criterion, not an assumed transmission distance and not a substitute for the 500 m regulatory separation applied to egg grading-and-packing facilities. For the event-level Cuzick–Edwards and season-specific analyses, all 204 layer and all 204 duck outbreak locations were retained as cases regardless of the nearest-farm distance. When a same-type registered farm was located within 1 km, that farm was removed once from the event-level control set so that a location could not contribute simultaneously as a case and a control. For the premises-level Bernoulli scans, only outbreaks within 1 km were assigned to a registered farm, and each successfully linked farm was classified as a case.

### 2.4. Case–Control Point-Pattern Construction

For Cuzick–Edwards and season-specific analyses, cases were outbreak events, and all 204 layer- and 204 duck-event locations were retained. Repeated events at the same location were retained as separate, coincident case points. Thus, premises affected in more than one epidemic season contributed one event in each affected season and more than one event to the overall point pattern. Registered farms of the same production type formed the control population; a farm linked within 1 km to one or more outbreak events was removed once from the control set. Outbreak events located more than 1 km from every same-type registered farm remained event-level cases but did not remove a registry farm from the control set. This design measures clustering of reported outbreak events and gives greater weight to recurrently affected locations; it should not be interpreted as a premises-level incidence analysis.

For the Bernoulli spatial scan, the unit of analysis was the distinct registered premises and case status was binary: a registered farm was classified as a case if at least one same-type outbreak location occurred within 1 km. Outbreak records outside the 1 km tolerance were not assigned to registered premises and therefore did not contribute farm-level case status. The Cuzick–Edwards analyses and Bernoulli scans consequently answer related but different questions—event aggregation versus premises-level excess risk. Separate point patterns were constructed for layer and duck farms, thereby normalizing each analysis to the spatial distribution of the corresponding production type.

### 2.5. Global Spatial Clustering Analysis

Event-level spatial clustering was quantified using the Cuzick–Edwards k-nearest-neighbor statistic [26]. The test evaluates whether case events have more neighboring case events among their k nearest points than expected under random labeling within the combined case–control point pattern. Because farm locations are retained during random labeling, the method accounts for the inhomogeneous spatial distribution of the production-type-specific farm background.

The statistic was evaluated at k = 3, 5, 7, and 10. These values were selected a priori to span very local neighborhoods and somewhat broader local structures and to avoid basing inference on a single neighborhood definition. Statistical significance was assessed with 999 Monte Carlo random-label permutations. For each production type and k value, the expected statistic, standard deviation, z-score, and Monte Carlo *p*-value were calculated. Layer and duck analyses used different background point patterns; therefore, their intensity ratios were compared descriptively and no direct between-type permutation test was performed.

Clustering intensity was summarized as the observed Cuzick–Edwards statistic divided by its permutation mean. Values greater than one indicate more neighboring case events than expected under random labeling. The ratio is a descriptive effect-size measure and no analytical confidence interval was available from the original output. Consistency across k values, z-scores, and Monte Carlo *p*-values was therefore emphasized rather than a single ratio.

### 2.6. Kernel Relative-Risk Surface and Spatial Scan Analysis

For layer farms, an exploratory kernel log relative-risk surface was estimated as the logarithm of the ratio between the smoothed event-case intensity and the smoothed control-farm intensity [28]. Following Kelsall and Diggle [28], a fixed, isotropic Gaussian kernel with a common bandwidth was applied to the numerator and denominator. The bandwidth was h = 10 km, selected by likelihood cross-validation on the combined layer case–control point pattern. A Silverman rule-of-thumb bandwidth of approximately 27.6 km was not used because it excessively smoothed the local structure of the nationwide point pattern. Positive values indicate areas where event intensity exceeded that expected from the layer-farm background. Because relative-risk surfaces depend on smoothing, the map was interpreted as exploratory and considered together with the formal scan statistic.

Premises-level clustering was localized with the Kulldorff Bernoulli spatial scan statistic [29]. Each registered farm was marked as a case if linked to at least one outbreak record. Candidate circular windows were restricted to a maximum radius of 50 km. This upper bound was chosen to permit detection of clusters spanning a regional poultry-production belt while preventing a candidate window from expanding across a large proportion of the country. For each window, the Bernoulli model compared the case proportion inside and outside the window. The most-likely cluster was identified by the maximum log-likelihood ratio, and significance was assessed with 199 Monte Carlo permutations. The scan was computationally intensive; 199 permutations yield a minimum attainable *p*-value of 0.005. Only the most-likely cluster was retained in the archived output, and secondary clusters were not evaluated.

For premises-level scanning, 188 of the 204 layer outbreak records were linked within 1 km to 147 distinct registered layer premises, and 160 of the 204 duck outbreak records were linked to 128 distinct registered duck premises. The remaining 16 layer and 44 duck records were retained in the event-level analyses but were not assigned to a registered premises for the Bernoulli scans. Analyses were conducted in R version 4.4.0 using spatstat version 3.1-1, sparr version 2.3-16, smacpod version 2.6.4, and smerc version 1.8.3 [30,31,32,33,34].

### 2.7. Spatiotemporal Trend

To evaluate temporal variation, the event-level Cuzick–Edwards statistic at k = 5 and the observed-to-expected intensity ratio were recomputed separately for each production type and epidemic season, using the fixed production-type-specific registry as the background. The k = 5 result was selected for seasonal presentation as an intermediate local neighborhood, while k = 3, 7, and 10 were retained for the overall sensitivity-to-neighborhood analysis. Because seasonal event counts varied substantially, seasonal ratios were interpreted descriptively. In particular, large ratios from seasons with few events may reflect the concentration of a small number of events and recurrent premises rather than a stable increase in underlying risk.

### 2.8. Space–Time Scan

A space–time permutation scan was applied to layer outbreak events using event location and epidemic season [35]. Candidate cylinders had circular spatial bases with a maximum radius of 50 km and consecutive-season temporal windows with a maximum length of three seasons. The three-season limit represented half of the six-season study period, allowing detection of persistent multi-season clustering without permitting a window that covered most of the observation period. Expected counts were conditioned on the spatial and temporal case margins. Significance was assessed by permuting season labels among event locations with 199 permutations. Only the most-likely space–time cluster was retained; secondary clusters were not available from the archived output.

## 3. Results

### 3.1. Distribution of Farms and Outbreaks

The dataset comprised 486 reported HPAI outbreak records between November 2020 and April 2026 and a fixed 2020 registry of 66,499 poultry premises. Outbreak records comprised 204 layer, 204 duck, and 78 other-poultry events. The registry included 1159 layer and 769 duck premises, which served as production-type-specific background populations. All 204 layer- and all 204 duck-event records entered the event-level analyses. For premises-level linkage, 188 layer records were within 1 km of a same-type registered farm and resolved to 147 distinct layer premises; 160 duck records were within 1 km and resolved to 128 distinct duck premises. Accordingly, 16 layer and 44 duck records were not assigned to registered premises for farm-level scanning. The median and 90th percentile of the nearest-registered-farm distance were 36 m and 0.69 km for layer events and 65 m and 2.83 km for duck events, respectively.

**Figure 1:**
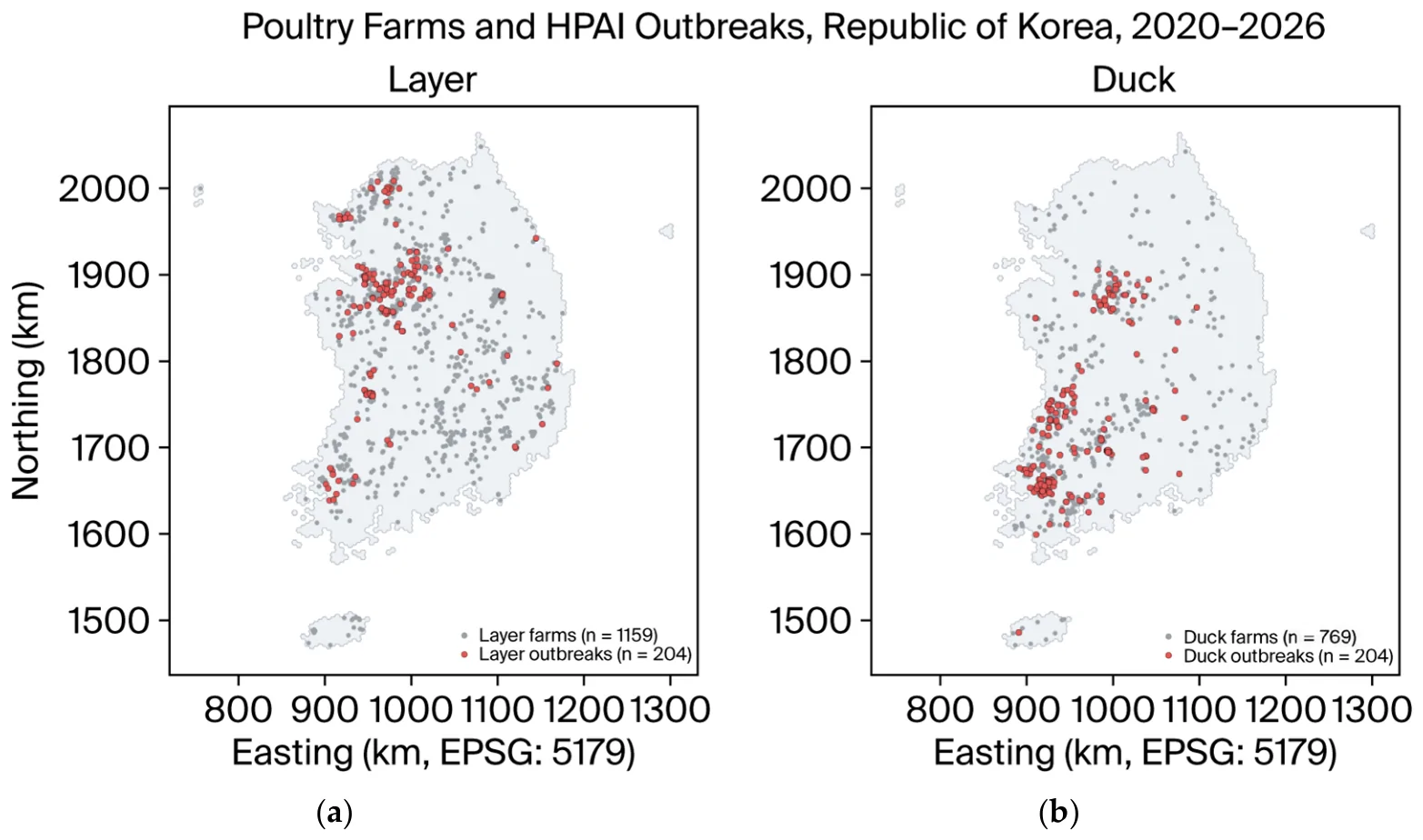
Spatial distribution of registered farms and HPAI outbreak events by production type in the Republic of Korea, 2020–2026. (**a**) Layer farms; (**b**) duck farms. Gray points indicate registered premises and red points indicate reported outbreak events; recurrent events may overlap at the same coordinate. Coordinates are displayed in the Korea 2000 Unified Coordinate System (EPSG:5179). Major layer- and duck-production concentrations occur in western and southwestern regions.

Layer and duck farms were both concentrated mainly in the western and southwestern regions of the ROK, where commercial poultry production is most intensive (Figure 1). However, the spatial distributions of the two production types were not identical, supporting the need for production-type-specific background populations rather than a pooled poultry-farm control population. Layer-farm outbreaks were observed within several layer-dense regions, whereas duck-farm outbreaks were more widely distributed across duck-producing areas.

### 3.2. Production-Type-Specific Global Clustering

Layer outbreak events showed significant local clustering relative to the layer-farm background. At k = 5, the observed Cuzick–Edwards statistic was 433, compared with a permutation expectation of 172.7, corresponding to a z-score of 16.98 and an observed-to-expected intensity ratio of 2.51 (*p* = 0.001; Table 2). This result indicates excess aggregation of reported layer outbreak events within the event-level point pattern.

Duck outbreak events also showed significant clustering. At k = 5, the observed statistic was 402 and the permutation expectation was 246.7, corresponding to a z-score of 9.14 and an intensity ratio of 1.63 (*p* = 0.001). Across k = 3, 5, 7, and 10, the layer ratios (2.42–2.57) were descriptively higher than the duck ratios (1.49–1.74). Because separate production-type backgrounds were used, this contrast was not a formal between-type hypothesis test.

### 3.3. Kernel Relative-Risk Surface

**Figure 2:**
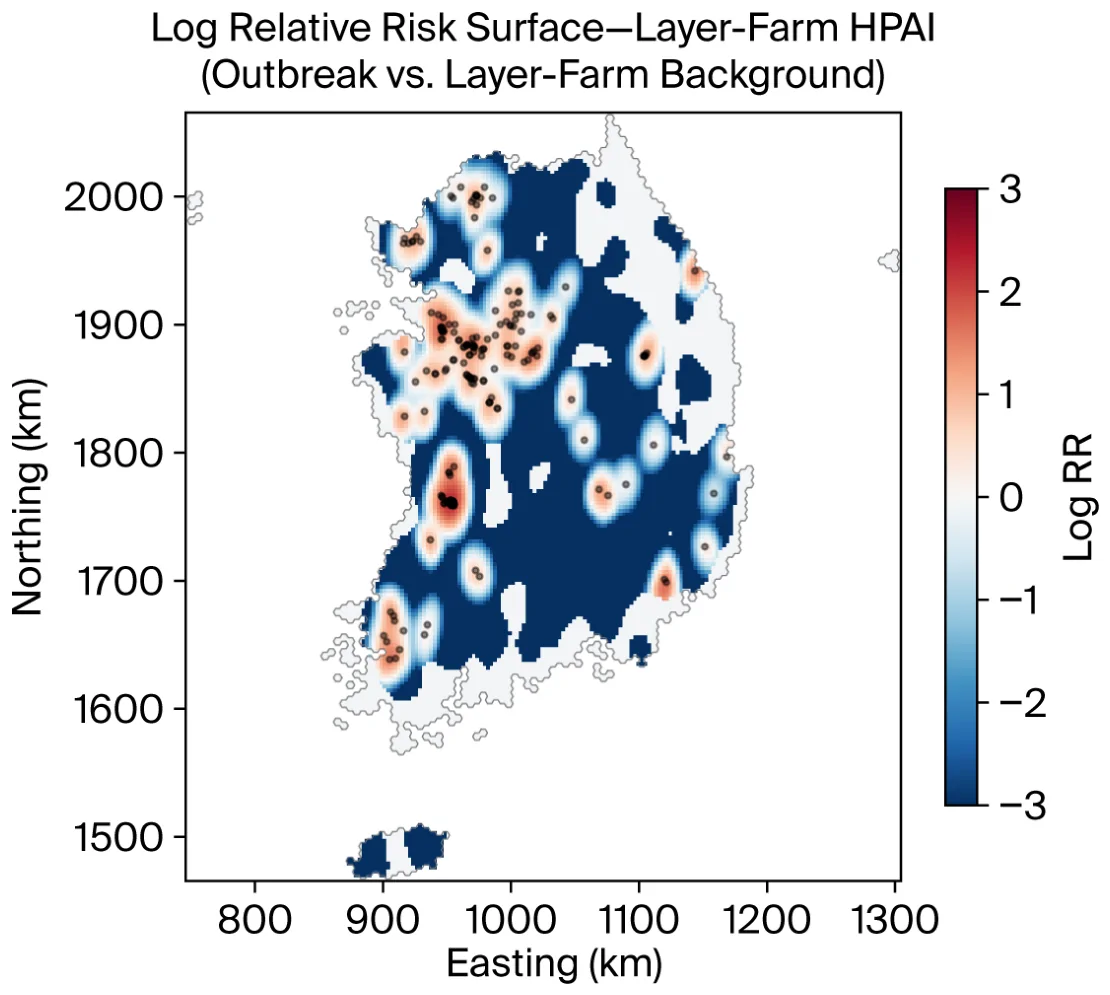
Exploratory kernel log relative-risk surface for layer-farm HPAI outbreak events in the Republic of Korea, 2020–2026. The surface is the log ratio of Gaussian-kernel-smoothed outbreak-event intensity to the registered layer-farm background intensity, estimated using a common fixed bandwidth of 10 km selected by likelihood cross-validation. Positive values indicate local excess event intensity after accounting for farm distribution. The result depends on the smoothing specification and is not a transmission map.

The exploratory kernel log relative-risk surface, estimated with a fixed isotropic Gaussian kernel and a common 10 km bandwidth, identified localized layer-production areas where outbreak-event intensity exceeded the smoothed layer-farm background (Figure 2). Positive values were concentrated in several western and west-central production regions. These areas were broadly consistent with the high-risk region identified by the premises-level spatial scan. The surface identifies areas for further investigation but does not distinguish farm-to-farm spread from shared exposure, recurrent introductions, or spatial differences in flock size, biosecurity, and surveillance.

### 3.4. Season-Specific Clustering

**Figure 3:**
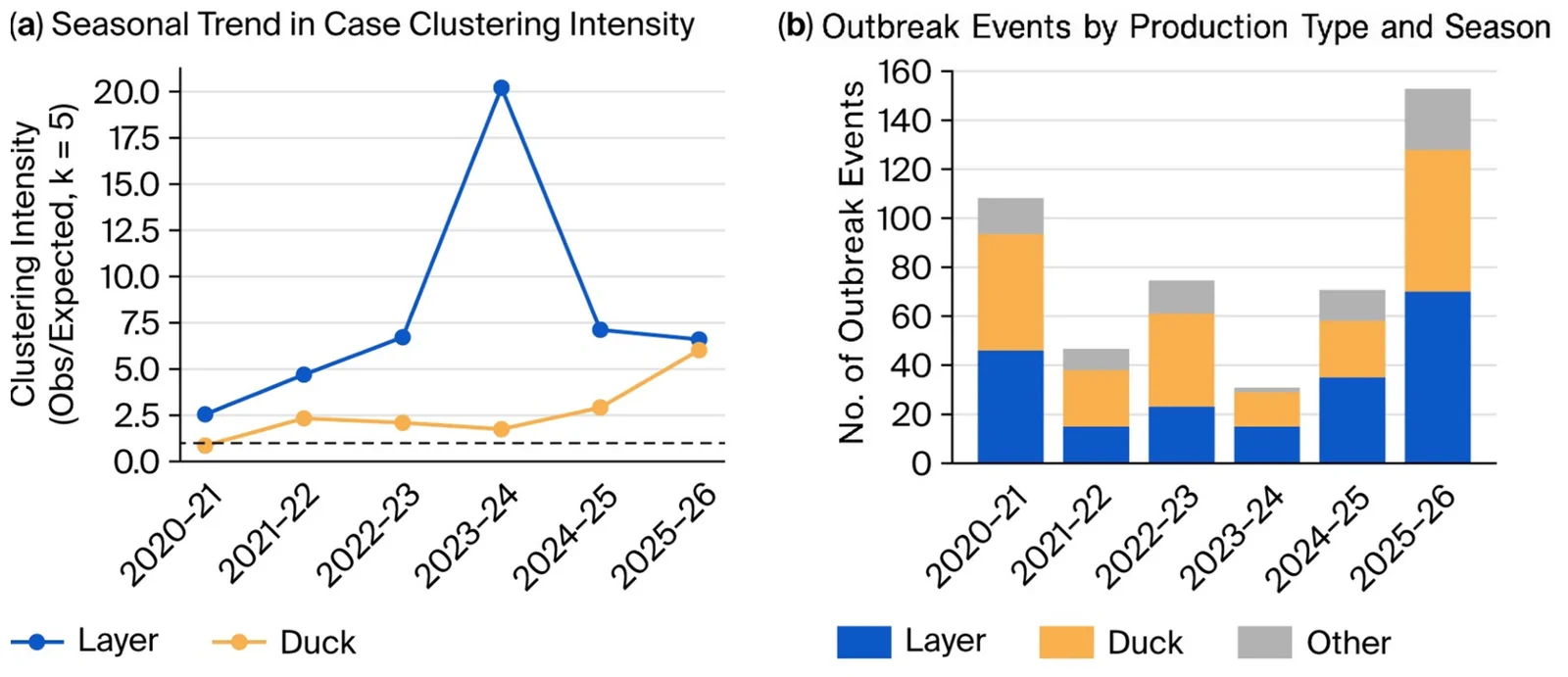
Season-specific HPAI event clustering and outbreak counts by production type. (**a**) Observed–to–expected Cuzick–Edwards intensity at k = 5 for layer and duck events. (**b**) Number of reported outbreak events by production type and epidemic season. The 2023–2024 layer estimate is based on 15 events and is sensitive to their local concentration.

Season-stratified analyses showed significant layer-event clustering in all six epidemic seasons (Table 3; Figure 3). The intensity ratios were 2.60, 4.74, 6.76, 20.23, 7.16, and 6.59 from 2020–2021 through 2025–2026, respectively. The exceptionally high 2023–2024 ratio was based on only 15 layer events. It reflects the strong concentration of a small event set relative to the fixed registry and may also be influenced by recurrent premises; it should not be interpreted as a twenty-fold increase in farm-level incidence. The largest number of layer events occurred in 2025–2026.

Duck-event clustering was more variable. It was not significant in 2020–2021 (intensity = 0.90, z = −0.34, *p* = 0.658) or 2023–2024 (intensity = 1.74, z = 0.60, *p* = 0.364), whereas significant clustering was observed in 2022–2023, 2024–2025, and 2025–2026. The largest duck ratio occurred in 2025–2026 (6.04; z = 18.74; *p* = 0.002). Seasonal ratios were interpreted together with event counts because sparse seasons can generate unstable observed-to-expected estimates.

### 3.5. Consistency Across Neighborhood Sizes

**Figure 4:**
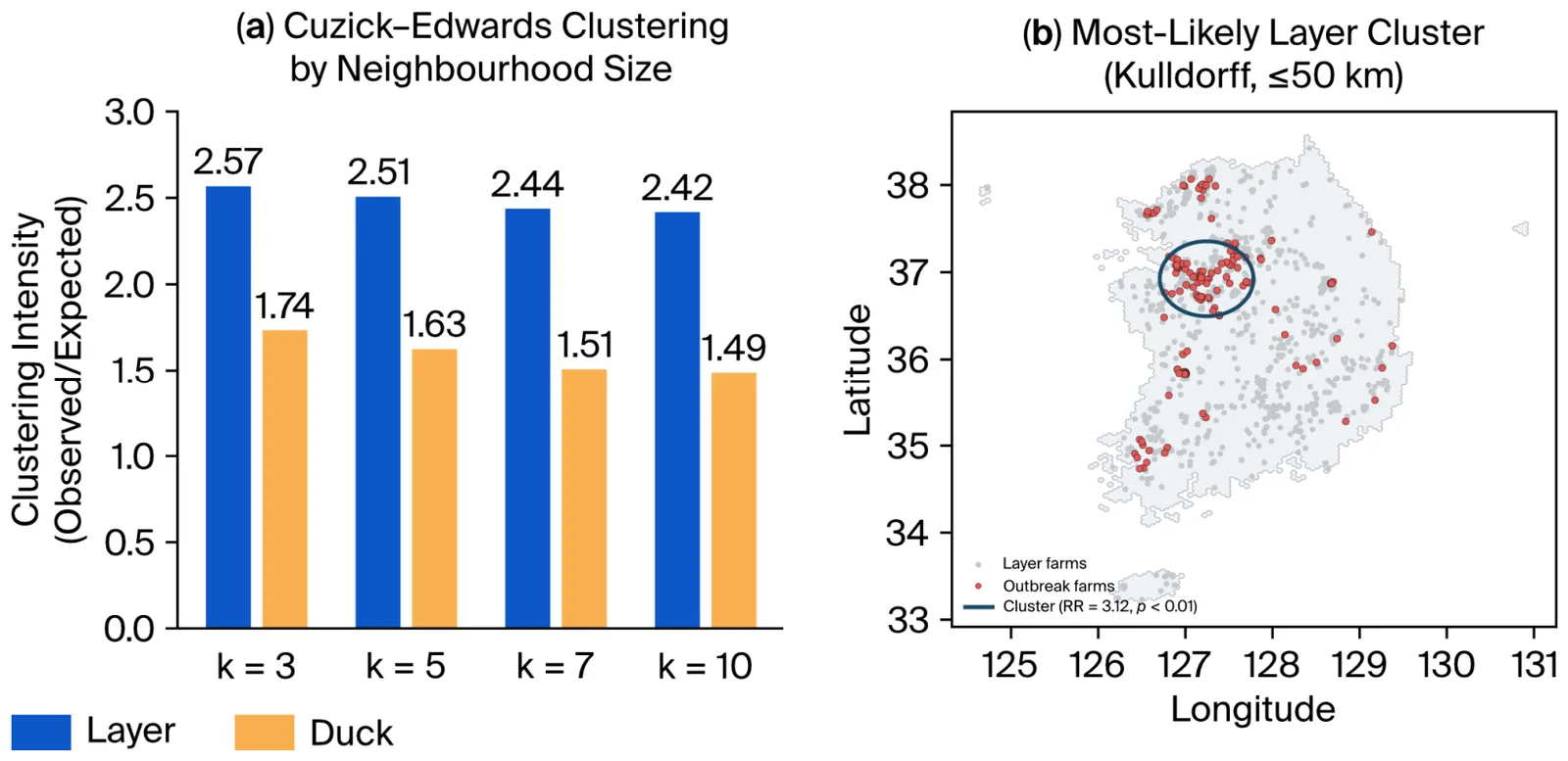
Cuzick–Edwards event-clustering intensity and most-likely layer-farm cluster. (**a**): Observed-to-expected event-clustering ratios by k for layer and duck analyses; the production-type contrast is descriptive. (**b**): Premises-level most-likely layer cluster from the Kulldorff Bernoulli scan with a 50 km maximum candidate radius. Gray points indicate registered layer farms, red points indicate outbreak premises, and the circle indicates the estimated cluster boundary.

The observed-to-expected clustering ratios were consistently higher for layer events than for duck events at k = 3, 5, 7, and 10 (Figure 4). Layer ratios decreased slightly from 2.57 at k = 3 to 2.42 at k = 10, whereas duck ratios decreased from 1.74 to 1.49. This pattern shows that the descriptive contrast was not dependent on a single neighborhood definition. It does not constitute a direct statistical test of the difference between production types.

### 3.6. Most-Likely Spatial Clusters

The premises-level Bernoulli spatial scan identified a most-likely layer cluster in the west-central ROK (Chungcheong), centered near 36.93° N, 127.24° E. The cluster radius was 47.7 km and included 240 registered layer farms, of which 66 were outbreak premises. The relative risk was 3.12, with a log-likelihood ratio of 25.6 and *p* < 0.01. The radius approached the pre-specified 50 km upper bound, so the boundary should be interpreted as a model-defined regional focus rather than a precise biological perimeter.

The most-likely duck cluster was much smaller: a 9.6 km radius containing 10 registered farms, of which 9 were outbreak premises. Its relative risk was 5.77, with a log-likelihood ratio of 13.4 and *p* < 0.01. Thus, the duck cluster had a higher local relative risk but involved only 10 farms, whereas the layer cluster covered a broad production region and included 240 farms. Relative-risk magnitude and geographic or operational scale should therefore be interpreted separately. Thus, the duck cluster had a higher local relative risk but involved only 10 farms, whereas the layer cluster covered a broad production region and included 240 farms. Relative-risk magnitude and geographic or operational scale should therefore be interpreted separately. The premises-level scan results are summarized in Table 4.

### 3.7. Space–Time Clustering of Layer-Farm Outbreaks

The space–time permutation scan identified a most-likely layer-event cluster in the west-central ROK, centered near 36.69° N, 127.17° E, with an 18.2 km radius during 2024–2025. The cylinder contained 12 events compared with an expectation of 2.6, producing a log-likelihood ratio of 9.3 and *p* = 0.005. This *p*-value is the minimum attainable value with 199 permutations.

**Figure 5:**
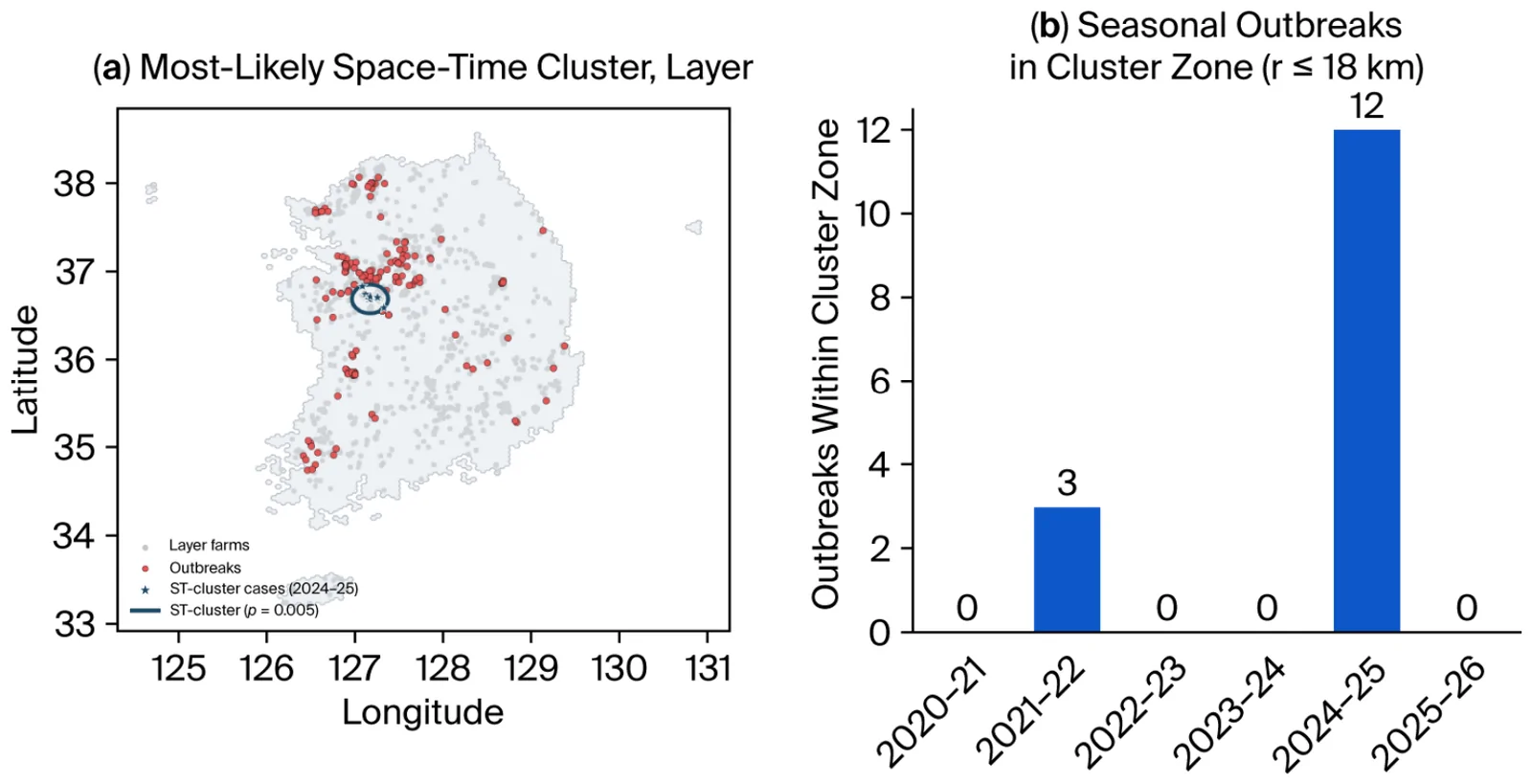
Most-likely space–time cluster of layer-farm HPAI outbreak events from the space–time permutation scan. (**a**) Estimated cluster location; the circle is the model-defined spatial window and stars are layer events during the cluster season. (**b**) Seasonal event counts within the 18.2 km zone. The most-likely cluster occurred during 2024–2025. Secondary clusters were not evaluated.

Within the cluster zone, events were concentrated in 2024–2025 and were uncommon in adjacent seasons. The result indicates localized temporal amplification, but the analysis did not incorporate viral sequence data, movements, environmental conditions, flock size, or changes in surveillance. Secondary space–time clusters were not retained in the archived output. The most-likely space–time cluster and seasonal event counts within the 18.2 km zone are shown in Figure 5. 

## 4. Discussion

This study used complementary event-level and premises-level spatial methods to evaluate HPAI clustering in layer and duck production. Both production types showed excess local aggregation relative to their own farm backgrounds, and the observed-to-expected event-clustering ratios were consistently higher in the layer analyses. However, the layer and duck point patterns had different background populations and no direct between-type inferential test was performed. The principal conclusion is therefore that layer outbreak events displayed a higher and more seasonally consistent descriptive clustering pattern, not that a statistically proven biological difference exists between host types.

Accounting for the farm population is a major strength of the design. Apparent concentrations of cases can arise simply because poultry farms are concentrated in production belts. The Cuzick–Edwards random-labeling framework and the case–control relative-risk surface normalized outbreak aggregation to farms of the same production type. Similar studies in France, the Netherlands, the United States, and Korea have linked HPAI risk or spread to farm density and proximity [7,8,12,15,16]. The current findings extend that literature by showing that recent Korean outbreak events remained locally aggregated even after conditioning on the broad spatial pattern of layer and duck farms.

The mechanisms underlying the layer pattern cannot be resolved from coordinates alone. Large layer operations may have larger workforces and more frequent feed, egg, manure, maintenance, and veterinary movements. In Japan, farms with at least 100,000 laying hens had increased outbreak risk during the 2022–2023 H5N1 epidemic [22]. Field and genomic investigations in Canada showed that suspected local transmission networks require integration of contact tracing and viral sequences rather than inference from proximity alone [18]. These findings support flock size and service connectivity as plausible explanations, but they do not identify any specific network in the Korean dataset.

The production-type contrast should also not be attributed to greater biological transmissibility in chickens. Experimental clade 2.3.4.4b H5N1 infection produced efficient duck-to-duck transmission, prolonged shedding, and extensive environmental contamination, whereas transmission among chickens was inefficient under the conditions tested [21]. Duck production can be influenced by wild-bird-associated exposure, but many commercial duck farms are enclosed and incursion can occur without direct wild-bird contact. The relative importance of wild-bird introduction, farm-to-farm movement, density, housing, and surveillance likely changes among seasons and production systems.

The 2023–2024 layer ratio of 20.23 requires particular caution. Only 15 layer events occurred in that season, and an observed-to-expected ratio can become very large when a small number of events are concentrated within a limited group of neighboring farms. Repeated events at the same premises also receive event-level weight in the Cuzick–Edwards statistic. The estimate therefore describes an unusually concentrated event pattern, not a twenty-fold increase in farm risk, and should not be used as evidence of a monotonic temporal trend. The largest layer-event count occurred in 2025–2026, when the ratio was lower but still elevated.

The premises-level scan statistics highlight a different distinction. The duck cluster had the higher local relative risk, but it contained only 10 farms, whereas the layer cluster encompassed 240 farms within a broad regional production belt. A small cluster can have a high relative risk when most farms inside the window are cases, while a larger cluster may be more important for regional preparedness despite a lower ratio. The kernel surface, global event-clustering statistic, Bernoulli scan, and space–time scan therefore describe different aspects of spatial structure and should not be interpreted as interchangeable estimates.

The 2024–2025 layer space–time cluster indicates localized seasonal amplification rather than a persistent national hotspot. Potential explanations include one or more viral introductions, local service contacts, environmental conditions, changes in surveillance, or chance concentration within a dense production area. Recent Korean molecular studies documented multiple H5N1, H5N6, and H5N3 clade 2.3.4.4b viruses across the 2023–2024 and 2024–2025 seasons [11], underscoring that spatially close outbreaks need not belong to a single transmission chain. Without original sequence data, the present study cannot separate lateral spread from independent introductions.

Egg-handling and grading infrastructure is one plausible component of layer-farm connectivity, but it was not directly evaluated. The study contained no coordinates for grading-and-packing facilities, egg collection routes, vehicle-sharing records, or facility-specific exposure measures. Accordingly, the results neither attribute clustering to these facilities nor evaluate the adequacy of the current 500 m separation requirement. They support only the broader proposition that density and service connectivity should be considered when designing surveillance and future facility-network studies.

Several potential confounders were not included. These include flock size, farm-level biosecurity, poultry and vehicle movements, egg collection networks, wild-bird density and infection status, wetlands and other environmental variables, surveillance intensity, and viral genotype. The registry categories also combined meat, breeder, and layer duck operations, which differ in production cycle, movements, housing, and biosecurity. These factors could account for part of the observed spatial and temporal heterogeneity and should be integrated in future multivariable or network-based analyses.

Additional methodological limitations warrant emphasis. First, the fixed October 2020 registry could misclassify the population at risk in later seasons because of farm opening, closure, relocation, changes in production type, and flock-size changes; differential turnover could bias the layer-duck contrast. Second, the 1 km linkage tolerance may assign an event to an incorrect same-type farm in dense areas, although it was used only to reconcile administrative coordinates and not as a transmission radius. Sixteen layer and 44 duck outbreak records were outside this tolerance and therefore contributed only to the event-level analyses, not to premises-level case status. The higher unmatched fraction and longer upper-tail matching distances for duck events may reflect registry turnover, coordinate discrepancies, or production changes, but the available data cannot distinguish among these explanations. Third, retaining recurrent events as coincident case points can increase event-level clustering ratios; the premises-level scan partly addresses this by using binary farm status. Fourth, the kernel surface used a fixed 10 km Gaussian bandwidth selected by likelihood cross-validation, but the resulting surface remains smoothing-dependent, and the scan results depend on the 50 km and three-season windows. Fifth, 199 scan permutations limited *p*-value resolution, secondary clusters were not retained, and no confidence intervals were available for the descriptive intensity ratios. These constraints reinforce the need to interpret clusters as hypothesis-generating areas rather than fixed transmission boundaries.

## 5. Conclusions

This national analysis demonstrates that reported HPAI events were locally aggregated relative to production-type-specific farm backgrounds and that the descriptive layer-event pattern was higher and more seasonally consistent than the duck-event pattern. Premises-level scans identified a broad layer-production cluster and a small high-relative-risk duck cluster, while the layer space–time analysis identified localized amplification in 2024–2025. These results support production-type-specific, density-aware surveillance but do not identify transmission pathways or implicate particular facilities. Validation with time-updated registries, distinct-premises sensitivity analyses, flock size, movement and service networks, biosecurity, wild-bird and environmental data, and viral sequences is required before causal or policy-specific conclusions are drawn.

## Figures and Tables

**Table 1:** Data sources and spatial preprocessing used in the analysis of HPAI outbreak clustering in poultry farms, Republic of Korea, 2020–2026.

Dataset	Source	Period	Size	Use in Analysis
HPAI outbreak records	KAHIS	Nov 2020–Apr 2026	486 events (layer 204; duck 204; other 78)	Event cases
Poultry-farm registry	Competent authority	1 Oct 2020	66,499 farms (layer 1159; duck 769)	Fixed controls (at-risk premises)
Coordinates	KAHIS/registry	—	WGS84 → EPSG:5179	Distance computation

Note: HPAI, high-pathogenicity avian influenza; KAHIS, Korea Animal Health Integrated System; EPSG:5179, Korea 2000 Unified Coordinate System. The registry provided the fixed 2020 background population for the unmatched case–control point-pattern analyses. Baseline flock size was available but was not modeled because season-specific updates were unavailable.

**Table 2:** Event-level spatial clustering of HPAI outbreak records relative to the same-type farm population by production type, Republic of Korea, 2020–2026.

Production Type	k	Observed	Expected	z	*p*	Intensity (Obs/Exp)
Layer	3	271	105.3	14.49	0.001	2.57
Layer	5	433	172.7	16.98	0.001	2.51
Layer	7	587	240.3	18.87	0.001	2.44
Layer	10	829	342.6	21.91	0.001	2.42
Duck	3	258	148.1	8.52	0.001	1.74
Duck	5	402	246.7	9.14	0.001	1.63
Duck	7	522	345.5	8.82	0.001	1.51
Duck	10	730	490.6	9.95	0.001	1.49

Note: Event clustering was evaluated with the Cuzick–Edwards k-nearest-neighbor statistic. Repeated outbreaks at the same premises were retained as separate event points. The expected value is the permutation mean under random labeling of event cases and control farms. Intensity is observed/expected. Statistical significance was assessed with 999 Monte Carlo permutations. Layer and duck ratios were not directly tested against one another.

**Table 3:** Season-specific event-level spatial clustering of HPAI outbreak records by production type, Republic of Korea, 2020–2026.

Season	Layer *n*	Layer Intensity	Layer *z*	Layer *p*	Duck *n*	Duck Intensity	Duck *z*	Duck *p*
2020–2021	46	2.60	3.88	0.002	48	0.90	−0.34	0.658
2021–2022	15	4.74	2.63	0.034	23	2.35	1.84	0.070
2022–2023	23	6.76	6.23	0.002	38	2.10	2.53	0.022
2023–2024	15	20.23	14.67	0.002	14	1.74	0.60	0.364
2024–2025	35	7.16	11.23	0.002	23	2.96	2.84	0.014
2025–2026	70	6.59	20.12	0.002	58	6.04	18.74	0.002

Note: Clustering was evaluated with the event-level Cuzick–Edwards statistic at k = 5. n is the number of outbreak events in each epidemic season, not necessarily the number of distinct premises. Intensity is observed/permutation expected. Ratios from seasons with small n should be interpreted cautiously.

**Table 4:** Most-likely spatial clusters of HPAI outbreaks by production type, identified using the Kulldorff Bernoulli spatial scan statistic.

Production Type	Radius (km)	Farms in Cluster	Outbreak Premises in Cluster	Relative Risk	Log-Likelihood Ratio	*p*
Layer	47.7	240	66	3.12	25.6	<0.01
Duck	9.6	10	9	5.77	13.4	<0.01

Note: The Bernoulli analysis used distinct registered premises, a maximum candidate radius of 50 km, and 199 Monte Carlo permutations. Farms in cluster is the number of registered premises; outbreaks in cluster is the number of premises with at least one matched outbreak. Only the most-likely cluster was retained, and secondary clusters were not evaluated.

## Data Availability

HPAI outbreak data are available from the Korea Animal Health Integrated System (KAHIS). The national poultry-farm registry was obtained under restricted access from the competent authority and cannot be shared publicly because it contains confidential premises information. The archived analysis code is available from the corresponding author on reasonable request; public release with example coordinates was not undertaken because the original workflow depends on restricted farm-location data.
